# Management of generalized anxiety disorder and panic disorder in general health care settings: new WHO recommendations

**DOI:** 10.1002/wps.21172

**Published:** 2024-01-12

**Authors:** Brandon Gray, Biksegn Asrat, Elaine Brohan, Neerja Chowdhury, Tarun Dua, Mark van Ommeren

**Affiliations:** ^1^ Department of Mental Health and Substance Use World Health Organization Geneva Switzerland; ^2^ Department of Psychiatry, Medicine and Health Sciences University of Gondar Gondar Ethiopia; ^3^ UK Public Health Rapid Support Team UK Health Security Agency/London School of Hygiene and Tropical Medicine London UK; ^4^ Centre for Global Mental Health London School of Hygiene and Tropical Medicine London UK

Mental, neurological and substance use (MNS) disorders are highly prevalent and account for a significant burden of disease^1^. However, in many countries, there is a gap between the need for MNS services and the available health system capacity and resources. The World Health Organization (WHO)'s mhGAP Action Programme was launched to address this gap by developing recommendations for the identification and management of priority MNS conditions in non‐specialist care settings. Several derivative tools, such as the mhGAP Intervention Guide (mhGAP‐IG), have been developed to support the programme's implementation^2^.

The mhGAP approach consists of interventions for the management of priority MNS conditions. These interventions are identified on the basis of evidence about their effectiveness and the feasibility of their scaling up in low‐ and middle‐income countries. Among MNS conditions covered in the first and second iterations of the mhGAP‐IG were depression, psychoses, self‐harm/suicide, epilepsy, dementia, disorders due to substance use in adults, and mental and behavioural disorders in children and adolescents.

The need has emerged for additional guidance on conditions not covered in the programme. Among these are anxiety disorders, which as a group are the most common mental disorders worldwide, with over 300 million people, about 4% of the global population, living with an anxiety disorder as of 2019^3^. Anxiety disorders also represent the second leading cause of disability‐adjusted life years (DALYs) among mental and substance use disorders^4^, and carry a significant social and economic burden^5^. Moreover, anxiety disorders have an early onset, representing the most prevalent mental disorder among older adolescents overall (4.6%), and particularly among adolescent girls (5.5%)^3^.

Although there are many effective treatments available, as many as 75% of people with anxiety disorders do not receive any care globally^6^. To address this gap, the WHO has developed a new module, as part of the mhGAP guideline update released in November 2023, to provide recommendations for the management of anxiety disorders and promote broader implementation of evidence‐based interventions in low‐ and middle‐income countries. This module focuses on generalized anxiety disorder (GAD) and panic disorder (PD), selected due to their prevalence, their estimated burden, the likelihood of their presentation in general health care settings, and the availability of evidence on feasibility and effectiveness of interventions in non‐specialist care settings.

The new mhGAP anxiety recommendations have been developed according to the WHO's guideline development process^7^. A Guideline Development Group (GDG) was convened and was responsible for making recommendations based on systematic review and appraisal of available evidence. Seven PICO questions were identified based on expert consensus to guide evidence retrieval, review, synthesis and assessment using the Grading of Recommendations, Assessment, Development and Evaluation (GRADE) system^8^. Results were then reviewed by the GDG to produce recommendations^9^. The mhGAP anxiety guidelines address the role of psychological interventions, pharmacotherapies, stress management, physical exercise, and collaborative care for adults with GAD or PD.

The guidelines recommend brief structured psychological interventions based on principles of cognitive behavioural therapy (CBT) for adults with GAD and/or PD. Most available evidence on the psychological treatment of GAD actually regards CBT, with third‐wave CBT also frequently studied. Evidence indicates that guided self‐help is likely to be more effective than unguided self‐help, and that specialist delivered interventions are likely to be more effective than those provided by non‐specialists, while there appear to be minimal to no differences between digital and face‐to‐face interventions, and between individual and group modalities.

The guidelines also recommend the use of selective serotonin reuptake inhibitors (SSRIs) for GAD and PD, while tricyclic antidepressants (TCAs) are recommended only for PD in cases where SSRIs are unavailable. No specific differentiation in terms of effectiveness or adverse effects emerged among the reviewed SSRIs, including citalopram, escitalopram, fluoxetine, paroxetine and sertraline. There was insufficient evidence for the use of TCAs in a‐dults with GAD.

Stress management techniques, including relaxation and/or mindfulness training, are also recommended for adults with GAD and/or PD, as is engagement in structured physical exercise. The guidelines recommend against the use of benzodiazepines in the treatment of adults with GAD and/or PD. These drugs should only be used for severe, acute anxiety symptoms and only as a very short‐term measure (3‐7 days). Finally, the guidelines recommend the consideration of collaborative care for adults with depression and/or anxiety and physical health conditions.

The GDG highlighted a number of key considerations in making these recommendations. First, the GDG emphasized that the WHO's process for guideline development does not intend to make recommendations that cover the totality of interventions proven effective in a given area^7^. Instead, the process focuses on areas or interventions where evidence is most substantial or where there have historically been controversies or the need for a policy change. Thus, the GDG noted that these initial guidelines may not encompass the totality of interventions that have been proven effective for GAD or PD.

Additionally, the GDG noted a limitation in the fact that the majority of evidence available comes from research conducted in high‐income countries, and highlighted the need for increased distribution of research funding to institutions in low‐ and middle‐income countries. It also noted considerable evidence for models of care, such as task‐sharing and training and supervision of non‐specialists, that are particularly appropriate for those countries. However, the GDG also specifically noted the challenges in human resources and health worker time and capacity to deliver certain interventions, particularly structured psychological interventions or collaborative care models.

Third, the GDG noted the need for further research to explore the longer‐term impact of interventions on symptoms, functioning and other key outcomes, while also recognizing the substantial evidence for symptom reduction in medium to short term. Fourth, the GDG made particular note of the need to consider cultural variability and individual preferences in applying recommendations in practice. For instance, the GDG highlighted the value of physical exercise for anxiety disorders generally, while also noting the need to consider daily habits of communities receiving care, such as when physical exertion is already a part of their daily life (e.g., farmers, manual labor workers).

Fifth, the GDG emphasized the need to ensure adequate training and follow‐up supervision for non‐specialists in any setting. Sixth, the GDG discussed the frequent over‐prescription of benzodiazepines for anxiety symptoms, particularly in non‐specialist care settings, and emphasized the risks associated with these prescriptions. Lastly, the GDG described the importance of adaptation for delivery of these interventions, including the use of innovative and digital technologies.

To date, there were no evidence‐based guidelines for managing common anxiety disorders in non‐specialized care settings focusing on low‐ and middle‐income countries. These recommendations were produced to fill this gap and will serve as a foundation for forming a new module in the mhGAP Intervention Guide, a tool frequently used to operationalize the mhGAP guidelines. Extensive work will be needed to scale up capacities in countries to act on these mhGAP recommendations and ensure effective management of anxiety disorders globally.
